# A CASE OF MULTIPLE SCLEROSİS PRESENTING AS DEPRESSION IN A YOUNG PERSON

**DOI:** 10.1192/j.eurpsy.2023.1765

**Published:** 2023-07-19

**Authors:** I. Sertler

**Affiliations:** Psychiatry, Erenkoy Mental and Neurological Diseases Education and Research Hospital, İstanbul, Türkiye

## Abstract

**Introduction:**

Multiple sclerosis(MS)is a chronic immune-mediated, inflammatory disease of the central nervous system. Depression is one of the most common psychiatric conditions associated with MS. Lifetime prevalence of major depression has been estimated to be around 50%.

**Objectives:**

Although the prevalence of depression in MS patients has been shown by many studies, there has not been a case in the literature demonstrating the onset of MS disease with depressive symptoms. In this article, a case of multiple sclerosis (undiagnosed before) presenting as depression in a young woman is presented and discussed.

**Methods:**

An eighteen years-old female patient visited the emergency room of Erenkoy Mental and Neurological Diseases Hospital with complaints of unhappiness,malaise,anhedonia,introversion,sleepiness,lack of appetite and nausea. She was admitted to another hospital one and a half years ago with similar complaints and duloxetine 30 mg/day treatment was started. The patient, who did not benefit from this treatment, stopped using the drug by herself and did not consult a doctor again. Her complaints regressed over time without treatment.Her current symptoms started four days prior to her visit. She had no other medically diagnosed condition. No pathology was detected in the emergency blood test parameters of the patient.Her brain tomography was normal. The patient, who had periventricular ovoid hyperintense lesions on cranial MRI, was diagnosed with multiple sclerosis and was admitted to the neurology unit of our hospital. The lumbar puncture was performed during the hospitalization, oligoclonal band positivity was detected. The IgG index was found to be increased at CSF (cerebrospinal fluid). She underwent pulse steroid therapy for seven days. The patient was discharged since her symptoms regressed after treatment.

**Results:**

Here we present a case of MS presenting as depression in a young woman. Depression is a very common, and very important comorbidity in multiple sclerosis. The etiology of depression in MS is likely to be multifactorial, and to include biological, psychological, and social determinants. Although the relationship between depression and MS has been clearly demonstrated in all of these studies, there is no case of MS presenting as depression in the literature.

**Conclusions:**

We know that depression may occur as a prodromal symptom in some organic diseases that affect brain such as dementia and Parkinson. In this case, we wanted to emphasize that organic etiological research is necessary for patients who present with psychiatric complaints for the first time, and that psychiatric complaints may be a symptom of organic diseases that may affect the brain.

**Disclosure of Interest:**

None Declared

